# Effects of Filtration Volumes on Bacterial Diversity and Community Structure in Freshwater Lakes

**DOI:** 10.1002/ece3.71445

**Published:** 2025-05-12

**Authors:** Chen Wang, Xinyu Chen, Zhen Shen, Jiaming Lv, Bobing Yu, Yansen Xu, Xiangming Tang

**Affiliations:** ^1^ College of Ecology and Applied Meteorology Nanjing University of Information Science & Technology Nanjing China; ^2^ Taihu Laboratory for Lake Ecosystem Research, State Key Laboratory of Lake Science and Environment Nanjing Institute of Geography and Limnology, Chinese Academy of Sciences Nanjing China; ^3^ College of Resources and Environment University of Chinese Academy of Sciences Beijing China; ^4^ College of Environment and Ecology Jiangnan University Wuxi China

**Keywords:** bacterial community structure, bacterial diversity, filtration volume, lake, membrane filtration

## Abstract

The use of membrane filtration is currently the most common method for collecting bacteria in lake water. However, the impact of different filtration volumes on bacterial diversity and community composition in lake water remains unclear. In this study, we collected water samples from mesotrophic Lake Bosten and eutrophic Lake Taihu in China. For Lake Bosten, we employed six filtration volumes (100, 200, 400, 800, 1600, and 3200 mL), while for Lake Taihu, seven filtration volumes (100, 200, 300, 400, 500, 1000, and 2000 mL) were used. Subsequently, Illumina MiSeq was employed to sequence the 16S rRNA genes, and statistical analyses were conducted on bacterial communities. Our study revealed that the water filtration volume impacts bacterial diversity, community structure, and taxonomic composition in both lakes. Based on the comprehensive consideration of the research results, we recommend using a filtering volume of 400–800 mL in mesotrophic lakes and 200–400 mL in eutrophic lakes for bacterial community studies.

## Graphical abstract

Our experimental results indicate that filtration volume affects bacterial diversity and taxonomic composition in lake ecosystems, with potentially more pronounced effects on mesotrophic lakes than eutrophic ones. For studies on bacterial communities in mesotrophic lakes, a filtering volume of 400–800 mL is recommended, whereas for eutrophic lakes, a volume of 200–400 mL is suggested. These recommended filtering volumes underscore the importance of using appropriate filtration volumes in comparative studies of bacterial communities in lakes with different nutrient levels.

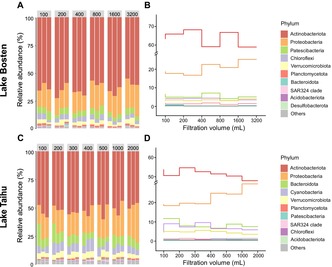

## 1 Introduction

In aquatic ecosystems, bacteria play a crucial role as key components of biogeochemical cycles. They are primary decomposers of organic matter (Cole et al. 1988; Cotner and Biddanda 2002) and have significant impacts on element fluxes and water quality in aquatic ecosystems (Pernthaler and Amann 2005). Lakes, as important wetland resources, play a vital role in the global carbon cycle (Cole et al. 2007) and are considered early indicators of global environmental and climate change (Adrian et al. 2009; Magnuson et al. 1990; Williamson et al. 2008). Currently, bacterial collection from lake water typically involves membrane filtration for subsequent research and analysis. However, the influence and biases of filtration water volume on bacterial diversity in lake water during membrane filtration are not well understood.

Most literature addressing the impact of filtration on bacterial diversity predominantly focuses on seawater as the research sample. Several studies have shown that different types of filters can affect marine bacterial activity, DNA extraction efficiency, and planktonic community structure (Djurhuus et al. 2017; Gasol and Morán 1999). Additionally, a few studies have indicated that water filtration volume may also influence DNA extraction efficiency and microbial community structure in seawater (Boström et al. 2004; Padilla et al. 2015). In contrast, studies on freshwater samples are relatively scarce. Less research has pointed out that filters with different pore sizes can affect bacterial diversity in lake water (Xie et al. 2020). Moreover, sample preparation methods and sample volume prior to DNA isolation (with the study only comparing different sample volumes when using eluate for DNA extraction) can also influence the results of next‐generation sequencing (Furtak et al. 2024).

Currently, there is substantial variability and inconsistency in the filtration volumes used for studying lake microbial communities. For instance, studies on microbial communities in eutrophic lakes use sample filtration volumes ranging from approximately 100 to 1000 mL (Lefranc et al. 2005; Yan et al. 2017; Ji et al. 2019; Xie et al. 2020). Similarly, studies on microbial communities in oligotrophic to mesotrophic lakes vary in sample filtration volumes, ranging from less than 100 to 1500 mL (Lefranc et al. 2005; Orellana et al. 2018; Richards et al. 2005; Xie et al. 2020). Some studies focusing on bacterial cultivation have filtration volumes as low as a few milliliters (Hahn et al. 2003), while others investigating microbial diversity in lakes of different nutrient states use consistent filtration volumes for both oligotrophic and eutrophic lakes (Zwirglmaier et al. 2015). The extent to which different filtering volumes affect bacterial community analysis is currently poorly understood. This leads to some uncertainty in the comparability of literature using different filtering volumes for bacterial diversity studies.

Theoretically and intuitively, bacterial species diversity should increase with a larger filtration volume because a larger volume is expected to contain rare taxa that smaller volumes might miss. However, the high‐throughput sequencing technology commonly used in bacterial diversity studies is based on PCR amplification. Amplification biases can favor the detection of dominant taxa, especially in larger volumes, which can overshadow rare species and artificially reduce the observed richness. Moreover, during the construction of clone libraries in high‐throughput sequencing, an equal amount of PCR amplification product is usually used for library construction and sequencing, meaning that not all of the initially extracted DNA is used in the final sequencing process. Therefore, a larger filtration volume may not necessarily result in higher observed bacterial species diversity. For example, in soil microbial studies, it has been shown that although sample volume can significantly affect microbial diversity and community structure, a larger sample volume does not necessarily guarantee higher species diversity (Christie and Beattie 1987; Ellingsøe and Johnsen 2002; Kang and Mills 2006; Li et al. 2023; Penton et al. 2016; Ranjard and Richaume 2001). Similarly, could this phenomenon also occur when using different filtration volumes in freshwater bacterial diversity studies? As the nutrient levels in the lake increase, the concentrations of nitrogen and phosphorus in the water gradually rise, leading to excessive algal blooms and making the water more difficult to filter (Shapiro 1980; Qin et al. 2006, 2013; Zhu et al. 2010). Therefore, in this study, we investigated the impact of filtration volume on bacterial diversity and community structure by applying different filtration volume gradients to samples from the mesotrophic Lake Bosten and the eutrophic Lake Taihu (Figure S1). We hypothesize that the filtering volume significantly influences bacterial diversity and community composition analysis results, and the optimal filtering volume varies between the two lakes.

## 2 Materials and Methods

### 2.1 Study Area, Sample Collection, and Filtration

Lake Bosten is located in the arid region of northwest China, characterized by a mesotrophic status, with a surface area of 1064 km^2^ and an average depth of 7.0 m. Prior to 1971, Lake Bosten was a freshwater lake. However, due to climate change and human activities, it transformed into an oligo‐saline lake with an average total dissolved solid (TDS) of 1.5 g/L. Since 2014, the water level has been rising, and by 2019, the TDS was measured to be less than 1 g/L, indicating a return to freshwater status (Tang et al. 2022). In this study, the physicochemical properties measured included a TDS concentration of 941 mg/L (Table S1). Lake Taihu, situated in the humid region of eastern China, is eutrophic with a surface area of 2238 km^2^ and an average depth of 1.9 m (Figure S2). Lake Bosten exhibits stratification in deeper areas of the water during the summer (with the deepest point around 16 m), but Lake Taihu does not.

In the summer of 2023, we collected epilimnetic water samples from Lake Bosten (41°53′10.94″ N, 86°50′45.93″ E) and Lake Taihu (31°25′7.92″ N, 120°12′47.61″ E) (sampling locations shown in Figure S2), approximately 0.5 m in depth. We collected five 10 L water samples from each lake's sampling point and within a 20‐m radius. The five samples from each lake were then thoroughly mixed to obtain a 50 L composite water sample for Lake Bosten and Lake Taihu, respectively. The field‐collected water samples were gathered using sterile containers and subsequently transferred to the laboratory. For Lake Bosten, subsamples were taken in volumes of 100, 200, 400, 800, 1600, and 3200 mL, while from Lake Taihu in volumes of 100, 200, 300, 400, 500, 1000, and 2000 mL. For each filtration volume, three replicates were determined: 18 samples from Lake Bosten and 21 samples from Lake Taihu, totaling 39 samples.

Subsequently, water samples were filtered through sterile 0.2 μm pore‐size polycarbonate membranes (Millipore, USA) using a vacuum filtration system. Prior to filtration, membranes were pre‐rinsed with sterile distilled water to minimize contamination. As per the experimental protocol, each sample was filtered with a predetermined volume through membrane filtration (additional membranes were used in case of membrane clogging). All membranes from the same sample are then combined into a single 2, 5, or 15 mL cryotube, depending on the number of membranes used for each sample, and stored at −80°C until DNA extraction.

### 2.2 Physical and Chemical Parameter Analysis

During the sample collection process, on‐site measurements of environmental factors were conducted at approximately 50 cm depth using a multi‐parameter water quality probe (YSI EXO2, Yellow Springs Instruments Inc., USA). The measured parameters included water temperature (WT), salinity (Sal), pH, and dissolved oxygen (DO). Transparency (SD) was determined using a Secchi disk. Standard methods were employed to measure total nitrogen (TN), total dissolved nitrogen (TDN), total phosphorus (TP), total dissolved phosphorus (TDP), chlorophyll‐*a* (Chl‐*a*), suspended solids (SS), suspended solids concentration (ISS), and loss on ignition (LOI) (Bridgewater et al. 2017).

### 2.3 Bacteria Counting

A volume of 1000 μL of the original water sample was mixed with 60 μL of formaldehyde (resulting in a final concentration of approximately 2%), and the mixture was fixed overnight at a constant temperature of 4°C. The following day, the fixed samples were transferred to −20°C for storage until bacterial counting. Prior to counting, SYBR Green I (Sigma‐Aldrich), diluted in dimethyl sulfoxide to a final concentration of 1:10,000, was added for staining. The staining process was maintained in the dark at room temperature. Yellow‐green fluorescent beads with a diameter of 1.0 μm were utilized as calibration and counting standards. Bacterial counting was performed using a flow cytometer (Becton Dickinson) according to previous literature (Gong et al. 2017).

### 2.4 DNA Extraction, PCR Amplification and Sequence Analysis

DNA extraction from the samples was carried out using the FastDNA Spin Kit for Soil (MP Biomedicals). According to the manufacturer's instructions, all membranes removed from the cryotube were carefully cut into small pieces and combined into a single sample for processing. The membranes were then mixed with reagents in the specified proportions to ensure optimal lysis and DNA recovery. The V3–V4 hypervariable region of the bacterial 16S rRNA gene was amplified using the primer set 338Fand 806R (Fadrosh et al. 2014). The PCR reactions were conducted as described earlier (Xie et al. 2021), and pair‐end sequencing was performed on the Illumina MiSeq platform (Illumina, USA) following the standard protocols of Guangdong Magigene Biotechnology Co. Ltd. (Shenzhen, China).

After demultiplexing, quality filtering, denoising, removing of chimeras, chloroplasts, and low‐abundance unique sequences (< 10 reads), we generated 2,776,914 high‐quality reads from the 18 samples of Lake Bosten (averaging 154,273 reads per sample) and 3,114,959 high‐quality reads from the 21 samples of Lake Taihu (averaging 148,331.4 reads per sample). The original sequences were analyzed using QIIME2 Core 2023.2 distribution (Bolyen et al. 2019), employing the DADA2 plugin (Callahan et al. 2016) for filtering, denoising, and quality control. This process yielded a feature table containing amplicon sequence variants (ASVs). Subsequently, the q2‐feature‐classifier plugin (Bokulich et al. 2018), utilizing a naive Bayes classifier based on the SILVA v138 database, was employed to classify representative sequences. ASVs with low abundance (< 10 reads) were filtered out to minimize random sequencing errors (Chao et al. 2024).

### 2.5 Statistical Analysis

This study conducted statistical analyses using R version 4.3.1. Data visualization was performed using the “ggplot2” and “vegan” packages in R software (Oksanen et al. 2009; Wilkinson 2011).

Bacterial α‐diversity indices, including Richness, Shannon, Faith phylogenetic diversity (Faith PD) and Evenness Index, were calculated for Lake Bosten and Lake Taihu based on minimum sequencing depths of 55,919 reads. To examine differences in bacterial α‐diversity among different filtration volumes, a non‐parametric Kruskal–Wallis test was conducted. Non‐metric multidimensional scaling (NMDS) based on Bray–Curtis distance was performed to visually display the differences in bacterial community structure between samples filtered at different volumes and among different replicates within the same filtering volume. The smaller the distance, the greater the similarity in community composition. Analysis of similarity (ANOSIM) and analysis of multivariate dispersion (ADONIS) were carried out to test differences among samples with different filtration volumes (Bai et al. 2022; Louca et al. 2016).

## 3 Results

### 3.1 Environmental Characterization

Characteristics of main environmental parameters and bacterial abundance are presented in Table S1. There were pronounced differences in nutrients between the two lakes. The TN concentration in Lake Taihu was approximately three times higher than that in Lake Bosten, and the TP concentration was 25 times higher in Lake Taihu compared to Lake Bosten. The concentrations of Chl‐*a* in Lake Taihu were about 50 times higher than that in Lake Bosten. The bacterial abundance in Lake Taihu was about 17 times higher than that in Lake Bosten.

### 3.2 Bacterial α‐Diversity Among Different Filtration Volumes

**FIGURE 1:**
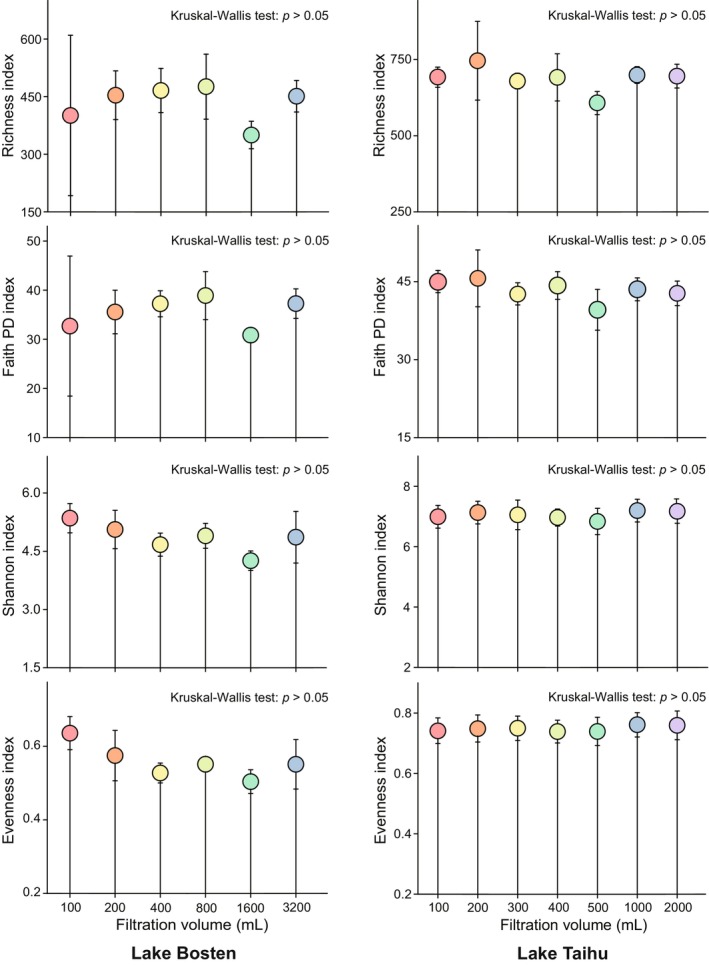
Comparison of the diversity indexes of Shannon, Richness, Faith PD, and Evenness in different filtered water volumes in Lake Bosten and Lake Taihu. The error bar represents the standard deviation among three parallel samples for each treatment. The Kruskal–Wallis test was used to check Figure 1 among the treatments, and Holm correction of *p*‐value correction by multiple comparisons was used. The *p*‐values of the statistical tests are all > 0.05, indicating that the α‐diversity did not change significantly among the different filtration volumes.

The rarefaction curves for Shannon, Faith PD, and Richness indices of α‐diversity for the two lakes are shown in Figure S3. As sequencing depth increased, the curves reached a saturation stage, indicating that the samples captured most of the bacterial species present in each filtered volume. The differences in α‐diversity indices among different filtration volumes were not statistically significant (Kruskal–Wallis test, *p* > 0.05) for both Lake Bosten and Lake Taihu, but subtle differences were observed (Figure 1). Specifically, with increasing filtration volume, the Richness and Faith PD indices showed a trend of initially increasing and then decreasing, with the maximum values observed at a filtration volume of 800 mL in Lake Bosten. The values of the Shannon and Evenness indices for bacteria exhibited a trend of initially decreasing and then increasing, with the maximum values observed at a filtration volume of 100 mL, and the samples filtered at 1600 mL had the lowest values for all four α‐diversity indices. Based on the rarefaction curves, we observed that the 800 mL samples in the Faith PD index reached saturation the latest, at a sequencing depth of approximately 40,000. In the Richness index, the 400 and 800 mL samples reached saturation the latest, at a sequencing depth of approximately 50,000 (Figure S3). In Lake Taihu, the values of the Richness and Faith PD indices for bacteria exhibited slight fluctuations with increasing filtration volume, reaching the maximum at a filtration volume of 200 mL. The values of the Shannon and Evenness indices were relatively uniform across all seven treatments, showing no apparent changes. Additionally, in the Faith PD index rarefaction curves, the 200 and 400 mL samples reached saturation the latest at a sequencing depth of approximately 40,000. In the Richness index rarefaction curves, the 400 mL sample reached saturation the latest, at a sequencing depth of around 45,000.

### 3.3 Bacterial β‐Diversity Among Different Filtration Volumes

**FIGURE 2:**
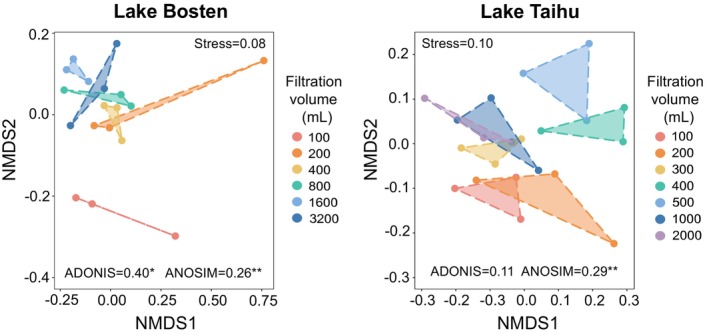
Nonmetric multidimensional scale (NMDS) of the distance between samples of Lake Bosten and Lake Taihu under different filtered water volumes. Analysis of similarity (ANOSIM) and analysis of multivariate dispersion (ADONIS) were used to examine differences among different filtration volumes.

The results of NMDS analysis, as well as ADONIS and ANOSIM tests, indicated that different volumes of filtered water had a certain impact on the bacterial community structure of both Lake Bosten and Lake Taihu (Figure 2). Specifically, in Lake Bosten, there were statistically significant differences in the bacterial community structure among samples filtered with six different volumes of water (*p* < 0.05). Samples filtered with a volume of 100 mL showed particularly large differences compared to the others. Samples filtered with a volume of 200 mL showed distinct variations within the triplicates. In Lake Taihu, the ADONIS results show that there were relatively small differences among the seven treatments (*p* > 0.05), but according to the ANOSIM analysis, there were still some features or variables that could distinguish the bacterial communities under different filtered volumes (*p* < 0.05).

### 3.4 Bacterial Taxonomy Among Different Filtration Volumes

**FIGURE 3:**
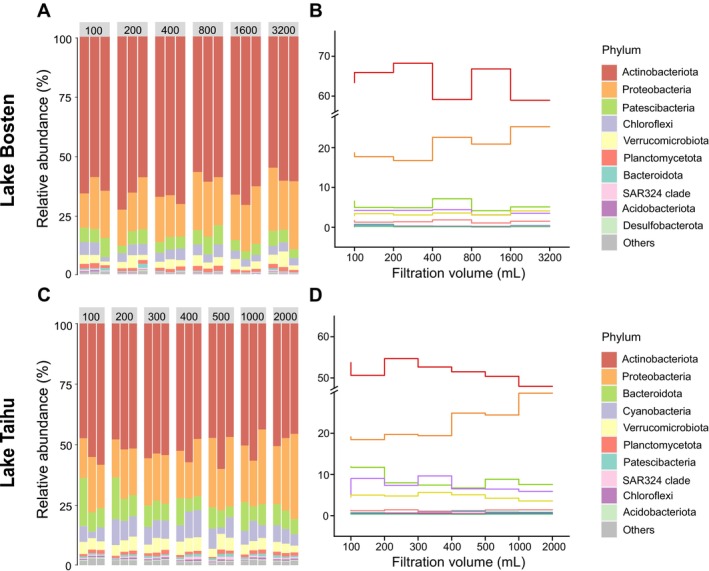
Relative abundance of major bacterial phyla in different filtration volumes (mL) of Lake Bosten (A) and Lake Taihu (C). The top 10 most abundant bacterial phyla are shown individually, with smaller taxa grouped as “other”. The vertical ladder diagram showed the change of relative abundance of the top 10 phyla under different filtration volumes in Lake Bosten (B) and Lake Taihu (D).

It was observed that in both lakes, the proportions of major bacterial phyla varied with increasing filtration volume (Figure 3). Specifically, in Lake Bosten, the top five bacterial phyla were Actinobacteria, Proteobacteria, Patescibacteria, Chloroflexi, and Verrucomicrobia (Figure 3A). The proportion of Actinobacteria was relatively smaller in samples filtered at 800 and 3200 mL. The proportion of Proteobacteria generally increased with the increase in filtration volume, while Patescibacteria had the highest proportion in the 800 mL sample and the lowest in the 1600 mL sample (Figure 3B). In Lake Taihu, the top five bacterial phyla were Actinobacteria, Proteobacteria, Bacteroidetes, Cyanobacteria, and Verrucomicrobia (Figure 3C). The proportion of Actinobacteria did not show significant changes with increasing filtration volume. The proportion of Proteobacteria tended to gradually increase, while the proportions of Bacteroidetes, Cyanobacteria, and Verrucomicrobia tended to decrease with increasing filtration volume (Figure 3D).

**FIGURE 4:**
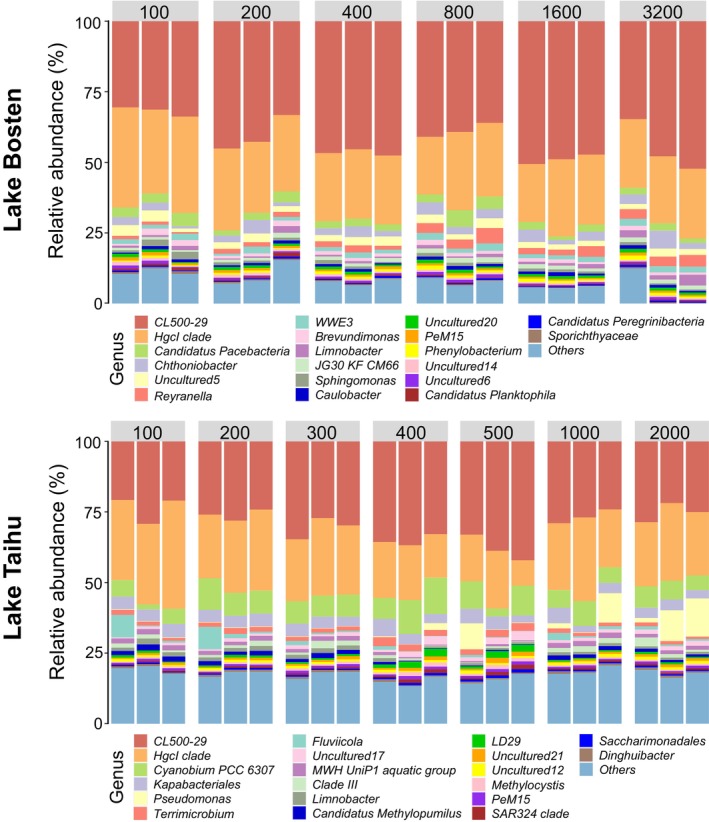
Relative abundance of major bacterial genera in different filtration volumes (mL) of Lake Bosten and Lake Taihu. The top 10 most abundant bacterial genera are shown individually, with smaller taxa grouped as “others”.

At the genus level (Figure 4), members of the Actinobacteria branch, including *CL500‐29* and *hgcI* clade, dominate in Lake Bosten. Moreover, we found that the proportion of *Reyranella*, which belongs to the α‐Proteobacteria, is very small when filtering water volumes are 100–200 mL, but gradually increases with increasing filtering volume, reaching its maximum proportion when the filtering volume is 800 mL, and then stabilizes. In Lake Taihu, when the filtering volume is 100–200 mL, there is a distinctly higher proportion of *Fluviicola* in one of the replicates compared to other treatments, while *Pseudomonas* gradually appears and occupies a certain proportion when the filtering volume ranges from 500 to 2000 mL.

Furthermore, the coefficient of variation (CV) indicates that when the filtration volume is between 400 and 800 mL, the CV of the top five bacterial phyla in Lake Bosten is relatively small, and the average CV value is also relatively low (Table 1). In Lake Taihu, the average CV of the dominant bacterial phyla is smallest when the filtering volume is 300 mL (Table 2). Additionally, the CVs of the dominant bacterial phyla are relatively small when the filtering volume ranges from 200 to 400 mL.

**TABLE 1:** Coefficient of variation (CV, %) and the mean values of the top five bacterial phyla with different filtration volumes in Lake Bosten.

Phylum	100	200	400	800	1600	3200
Actinobacteriota	5.8	10.4	2.9	3.5	5.9	5.5
Proteobacteria	20.5	20.2	16.5	9.7	13.6	16.5
Patescibacteria	20.6	33.2	7.8	15.2	16.9	25.5
Chloroflexi	48.5	11.4	13.4	21.6	13.2	11.8
Verrucomicrobiota	40.9	50.6	27.1	27.7	45.9	56.1
Mean	27.3	25.2	13.5	15.6	19.1	23.1

**TABLE 2:** Coefficient of variation (CV, %) and the mean values of the top five bacterial phyla with different filtration volumes in Lake Taihu.

Phylum	100	200	300	400	500	1000	2000
Actinobacteriota	10.5	4.5	1.8	9.2	14.6	12.8	5.4
Proteobacteria	17.5	13.6	1.8	22.5	26.6	24.7	18.9
Bacteroidota	57.6	41.2	12.4	44.1	32.6	33.7	16.3
Cyanobacteria	40.2	16.1	2.0	26.3	50.8	26.0	18.9
Verrucomicrobiota	17.3	14.6	26.0	1.1	31.6	12.3	18.8
Mean	28.6	18.0	8.8	20.6	31.2	21.9	15.7

## 4 Discussion

The results of this experiment indicate that the volume of filtered water affects bacterial diversity, community structure, and taxonomic composition in lakes with different nutrient levels. Here we will discuss which filtering volume range is most recommended for the study of bacterial communities in mesotrophic and eutrophic lakes.

### 4.1 Filtration Volumes Ranging From 400 to 800 mL Are Recommended for Bacterial Community Research in Mesotrophic Lakes

Our experimental results suggest that filtering water samples of 400–800 mL volume is more appropriate for studying bacterial diversity in mesotrophic lakes. In Lake Bosten, when filtration volumes are between 400–800 mL, the Richness and PD indices of α‐diversity are maximized, and Shannon and Evenness indices also rank high (Figure 1). Moreover, according to α‐diversity analysis, bacterial diversity is lower when the filtration volume is 1600 mL, indicating that there is not a positive correlation between filtering volume and bacterial diversity (Boström et al. 2004). Hence, larger filtering volumes are not necessarily better.

From the perspective of β‐diversity (Figure 2), when filtration volume is 400–800 mL, the differences in community among the replicates are relatively small, indicating reliable repeatability. In contrast, when the filtration volumes are 100 and 200 mL, significant differences exist in bacterial communities among replicates, suggesting greater dispersion and less representativeness. However, with filtering volumes ranging from 400 to 800 mL, the differences in bacterial communities among replicates and treatments diminish and tend to be consistent, better reflecting the bacterial community structure of Lake Bosten. This aligns well with the requirements for subsequent analysis.

In terms of taxonomic composition, filtration volumes of 400–800 mL are more suitable compared to other filtration volumes. At the phylum level, the dominant bacterial phyla in Lake Bosten are Actinobacteria and Proteobacteria, consistent with other related research results (Figure 3A) (Feng et al. 2019; Hugoni et al. 2017). Actinobacteria have been shown to encompass lineages with varying salinity preferences (Liu et al. 2015; Shen et al. 2018). Additionally, at the phylum level, taxa such as Patescibacteria, Chloroflexi, and Verrucomicrobiota, which have relatively low abundance, increase significantly when the filtering volume ranges from 400 to 800 mL (Figure 3B). At the genus level, *CL500‐29* and *hgcI clade* dominated in Lake Bosten (Figure 4). They are particularly inclined towards distribution in low salinity freshwater lakes, serving as strong indicators of oligo‐to‐mesohaline conditions (Mohapatra et al. 2020). This is consistent with the fact that Lake Bosten was previously in an oligo‐saline state (Tang et al. 2020, 2022). The genus *Reyranella* thrives in freshwater environments (Pagnier et al. 2011). In Lake Bosten, the relative abundance of Reyranella is relatively low when the filtration volume is less than 400 mL. As the filtration volume increases beyond 400 mL, the relative abundance of Reyranella rises and stabilizes. This suggests that a filtration volume between 400 and 800 mL is more suitable for capturing the structural characteristics of the bacterial community. Moreover, the small average coefficient of variation values obtained using the filtration volumes between 400 and 800 mL (Table 1) suggest that the bacterial community composition has lower dispersion and smaller differences, making it more suitable for bacterial diversity analysis. The data of filtered water samples with a filtration volume of more than 800 mL is also relatively good, but 400–800 mL water samples are sufficient to support the study of bacterial diversity, and the bacterial community structure is relatively stable, which greatly saves time and research costs. For example, Aguayo et al. (2017) chose 500 mL as the volume of filtered water sample when comparing the bacterial diversity of three oligotrophic lakes in Patagonia. Xing et al. (2020) selected 600 mL as the filtered water sample when studying stratification of microbiomes during the holomictic period of oligotrophic Lake Fuxian. Liu et al. (2015) chose filtered water samples ranging from 500 to 1000 mL when elucidating the response of bacterial communities in Lake Bosten to environmental changes. All three studies yielded relatively good results. Therefore, we recommend 400–800 mL of filtered water sample volume for the study of bacterial diversity in mesotrophic lakes. It seems that 800 mL filtration volume is also suitable for bacterial community study in oligotrophic lakes.

### 4.2 Filtration Volumes Ranging From 200 to 400 mL Are Recommended for Bacterial Community Research in Eutrophic Lakes

Our findings from Lake Taihu suggest that filtration samples with volumes ranging from 200 to 400 mL is more suitable for studying bacterial communities in eutrophic lakes. According to the results of α‐diversity analysis (Figure 1), the highest richness and Faith PD are observed when filtering water volumes range from 200 to 400 mL, although the differences among different filtering volumes are not significant.

From the perspective of β‐diversity (Figure 2), when the filtering volume is 300 mL, the internal differences among replicates are the smallest, indicating high repeatability of bacterial community structures. Furthermore, as the filtering volume gradually increases to 1000 and 2000 mL, the community structures become more similar to those from the 300 mL filtering volume. Therefore, it appears that a sample volume around 300 mL is preferable, reducing the time and cost of species analysis and making them more suitable for bacterial β‐diversity analysis.

In terms of taxonomic composition, the main bacterial phyla are Actinobacteria and Proteobacteria (Figure 3C), consistent with findings from eutrophic lakes such as Lake Donghu (Feng et al. 2019; Ji et al. 2019). Additionally, the proportions of bacterial phyla with relatively low abundance, such as Cyanobacteria, Verrucomicrobiota, and Planctomycetota, gradually increase and stabilize with the filtering volume ranging from 200 to 400 mL (Figure 3D). High filtering volumes (> 500 mL) can result in an increase in the abundance of certain rare bacterial genera, such as *Pseudomonas* (Figure 4). *Pseudomonas* species are typically uncommon in freshwater (Newton et al. 2011); however, as the filtered volume increases, the relative abundance of *Pseudomonas* also increases. This suggests that increasing the volume of filtered water makes it easier to detect some specific rare bacterial species. Furthermore, when the filtering volume ranges from 200 to 400 mL, the CVs are relatively small (Table 2), suggesting smaller differences and better representation in taxonomic composition compared to other treatments. This filtering volume range is also widely utilized in other studies. For example, Ren et al. (2019) opted for a volume of 200 mL when examining the functional characteristics of the bacterial community in a eutrophic river. Similarly, Xie et al. (2020) selected 300 mL as the filtered water sample volume from two eutrophic lakes to explore the impact of particle collection methods on bacterial diversity. Hu et al. (2020) used 400 mL as the filter water sample volume when investigating the relationship between free‐living and particle‐attached bacteria in mesotrophic to eutrophic Lake Wuli.

### 4.3 Species Richness and the Coefficient of Variation (CV) of Dominant Bacterial Phyla

We observed that species richness does not necessarily increase with larger volumes; for instance, in Lake Bosten, species richness actually decreased when the filtration volume exceeded 800 mL. We speculate that this may be due to two main reasons: First, the overrepresentation of dominant taxa in larger volumes, where dominant taxa are more likely to be captured in greater quantities, which can lead to their preferential amplification during sequencing (Penton et al. 2016). This amplification may obscure the detection of rare species, resulting in a lower observed richness (Gonzalez et al. 2012). Second, during high‐throughput sequencing, an equal amount of PCR product is typically used for library construction and sequencing, meaning not all extracted DNA is sequenced. Thus, larger sample volumes may not necessarily lead to higher observed bacterial diversity (Christie and Beattie 1987; Ellingsøe and Johnsen 2002; Kang and Mills 2006; Li et al. 2023; Penton et al. 2016; Ranjard and Richaume 2001). We also observed that the coefficients of variation (CV) of the dominant bacterial phyla in both lakes were relatively high outside the recommended filtration volumes. We speculate that the higher species CV in smaller filtration volumes is due to a greater susceptibility to random effects and sampling biases. With smaller filtration volumes, there is a higher likelihood of capturing only a small portion of the microbial community, leading to greater differences between replicates as random fluctuations play a more significant role (Forcino et al. 2015). In contrast, the higher CV observed in larger filtration volumes can be attributed to the increased probability of detecting low‐abundance species that were not captured in smaller volumes, potentially resulting in greater variability between samples (Turner et al. 2014; Forcino et al. 2015; Sepulveda et al. 2019).

Our experimental results also suggest that the impact of filtering volume on mesotrophic lakes may be more significant than that on eutrophic lakes. In the water samples from Lake Taihu, there is relatively little variation in the four indices of bacterial α‐diversity with increasing filtering volume, without a clear trend (Figure 1), whereas Lake Bosten exhibits more pronounced trends. The differences in bacterial community composition among different filtering volumes in Lake Taihu are minor, whereas significant differences exist in Lake Bosten, especially in the bacterial community composition when filtered at 100 and 200 mL compared to the other four filtering volumes (Figure 2). Additionally, the changes in the dominant bacterial phyla in Lake Taihu with different filtering volumes are smaller than those in Lake Bosten (Figure 3).

All samples were collected in August 2023 to minimize temporal variability. We also acknowledge the limitations of our experiment and recognize the importance of conducting repeat experiments using samples from more lakes with different nutrient levels. We emphasize that our study and findings are merely preliminary explorations. In the future, more extensive research, including a broader range of nutrient levels and increased replicates, is needed to ensure more comprehensive and reliable results.

## 5 Conclusions

Our experimental results indicate that filtration volume affects bacterial diversity and taxonomic composition in lake ecosystems, with potentially more pronounced effects on mesotrophic lakes than eutrophic ones. For studies on bacterial community in mesotrophic lakes, a filtering volume of 400–800 mL is recommended, whereas for eutrophic lakes, a volume of 200–400 mL is suggested. These recommended filtering volumes underscore the importance of using appropriate filtration volumes in comparative studies of bacterial communities in lakes with different nutrient levels.

## Author Contributions

**Chen Wang:** data curation (equal), formal analysis (equal), investigation (equal), software (equal), writing – original draft (equal). **Xinyu Chen:** formal analysis (equal), validation (equal), visualization (equal). **Zhen Shen:** formal analysis (equal), investigation (equal). **Jiaming Lv:** formal analysis (equal), resources (equal). **Bobing Yu:** investigation (equal), resources (equal). **Yansen Xu:** conceptualization (equal), investigation (equal), supervision (equal). **Xiangming Tang:** conceptualization (equal), funding acquisition (equal), methodology (equal), writing – review and editing (equal).

## Conflicts of Interest

The authors declare no conflicts of interest.

## Supporting information


Data S1

## Data Availability

The raw sequence data have been deposited in the Genome Sequence Archive (Genomics, Proteomics & Bioinformatics 2021) in the National Genomics Data Center (Nucleic Acids Res 2022), China National Center for Bioinformation/Beijing Institute of Genomics, Chinese Academy of Sciences (GSA: CRA015860) that are publicly accessible at https://ngdc.cncb.ac.cn/gsa.

## References

[ece371445-bib-0001] Adrian, R. , C. M. O'Reilly , H. Zagarese , et al. 2009. “Lakes as Sentinels of Climate Change.” Limnology and Oceanography 54: 2283–2297. 10.4319/lo.2009.54.6_part_2.2283. 20396409 PMC2854826

[ece371445-bib-0002] Aguayo, P. , P. González , V. Campos , et al. 2017. “Comparison of Prokaryotic Diversity in Cold, Oligotrophic Remote Lakes of Chilean Patagonia.” Current Microbiology 74: 598–613. 10.1007/s00284-017-1209-y. 28265709

[ece371445-bib-0003] Bai, C. , G. Gao , X. Tang , et al. 2022. “Contrasting Diversity Patterns and Community Assembly Mechanisms of Bacterioplankton Among Different Aquatic Habitats in Lake Taihu, a Large Eutrophic Shallow Lake in China.” Environmental Pollution 315: 120342. 10.1016/j.envpol.2022.120342. 36240961

[ece371445-bib-0004] Bokulich, N. A. , B. D. Kaehler , J. R. Rideout , et al. 2018. “Optimizing Taxonomic Classification of Marker‐Gene Amplicon Sequences With QIIME 2's q2‐Feature‐Classifier Plugin.” Microbiome 6: 90. 10.1186/s40168-018-0470-z. 29773078 PMC5956843

[ece371445-bib-0005] Bolyen, E. , J. R. Rideout , M. R. Dillon , et al. 2019. “Reproducible, Interactive, Scalable and Extensible Microbiome Data Science Using QIIME 2.” Nature Biotechnology 37, no. 8: 852–857. 10.1038/s41587-019-0209-9. PMC7015180 31341288

[ece371445-bib-0006] Boström, K. H. , K. Simu , Å. Hagström , and L. Riemann . 2004. “Optimization of DNA Extraction for Quantitative Marine Bacterioplankton Community Analysis.” Limnology and Oceanography: Methods 2: 365–373. 10.4319/lom.2004.2.365.

[ece371445-bib-0007] Bridgewater, L. L. , R. B. Baird , A. D. Eaton , et al., eds. 2017. Standard Methods for the Examination of Water and Wastewater. 23rd ed. American Public Health Association.

[ece371445-bib-0008] Callahan, B. J. , P. J. McMurdie , M. J. Rosen , A. W. Han , A. J. A. Johnson , and S. P. Holmes . 2016. “DADA2: High‐Resolution Sample Inference From Illumina Amplicon Data.” Nature Methods 13: 581–583. 10.1038/nmeth.3869. 27214047 PMC4927377

[ece371445-bib-0009] Chao, J. , J. Li , M. Kong , K. Shao , and X. Tang . 2024. “Bacterioplankton Diversity and Potential Health Risks in Volcanic Lakes: A Study From Arxan Geopark, China.” Environmental Pollution 342: 123058. 10.1016/j.envpol.2023.123058. 38042466

[ece371445-bib-0010] Christie, P. , and J. A. M. Beattie . 1987. “Significance of Sample Size in Measurement of Soil Microbial Biomass by the Chloroform Fumigation‐Incubation Method.” Soil Biology and Biochemistry 19: 149–152. 10.1016/0038-0717(87)90074-5.

[ece371445-bib-0011] Cole, J. , S. Findlay , and M. Pace . 1988. “Bacterial Production in Fresh and Saltwater Ecosystems: A Cross‐System Overview.” Marine Ecology Progress Series 43: 1–10. 10.3354/meps043001.

[ece371445-bib-0012] Cole, J. J. , Y. T. Prairie , N. F. Caraco , et al. 2007. “Plumbing the Global Carbon Cycle: Integrating Inland Waters Into the Terrestrial Carbon Budget.” Ecosystems 10: 172–185. 10.1007/s10021-006-9013-8.

[ece371445-bib-0013] Cotner, J. B. , and B. A. Biddanda . 2002. “Small Players, Large Role: Microbial Influence on Biogeochemical Processes in Pelagic Aquatic Ecosystems.” Ecosystems 5: 105–121. 10.1007/s10021-001-0059-3.

[ece371445-bib-0014] Djurhuus, A. , J. Port , C. J. Closek , et al. 2017. “Evaluation of Filtration and DNA Extraction Methods for Environmental DNA Biodiversity Assessments Across Multiple Trophic Levels.” Frontiers in Marine Science 4: 314. 10.3389/fmars.2017.00314.

[ece371445-bib-0015] Ellingsøe, P. , and K. Johnsen . 2002. “Influence of Soil Sample Sizes on the Assessment of Bacterial Community Structure.” Soil Biology and Biochemistry 34: 1701–1707. 10.1016/S0038-0717(02)00156-6.

[ece371445-bib-0016] Fadrosh, D. W. , B. Ma , P. Gajer , et al. 2014. “An Improved Dual‐Indexing Approach for Multiplexed 16S rRNA Gene Sequencing on the Illumina MiSeq Platform.” Microbiome 2: 6. 10.1186/2049-2618-2-6. 24558975 PMC3940169

[ece371445-bib-0017] Feng, C. , J. Jia , C. Wang , et al. 2019. “Phytoplankton and Bacterial Community Structure in Two Chinese Lakes of Different Trophic Status.” Microorganisms 7: 621. 10.3390/microorganisms7120621. 31783682 PMC6956004

[ece371445-bib-0018] Forcino, F. L. , L. R. Leighton , P. Twerdy , and J. F. Cahill . 2015. “Reexamining Sample Size Requirements for Multivariate, Abundance‐Based Community Research: When Resources Are Limited, the Research Does Not Have to Be.” PLoS One 10: e0128379. 10.1371/journal.pone.0128379. 26058066 PMC4461312

[ece371445-bib-0019] Furtak, K. , A. Marzec‐Grządziel , and M. S. Hossain . 2024. “Pre‐Isolation Procedures Matter–Comparison of Different Filtration Methods Prior to DNA Isolation in River Microbiome Analysis.” Ecohydrology and Hydrobiology 24: 486–491. 10.1016/j.ecohyd.2023.12.004.

[ece371445-bib-0020] Gasol, J. M. , and X. A. G. Morán . 1999. “Effects of Filtration on Bacterial Activity and Picoplankton Community Structure as Assessed by Flow Cytometry.” Aquatic Microbial Ecology 16: 251–264. 10.3354/ame016251.

[ece371445-bib-0021] Gong, Y. , X. Tang , K. Shao , Y. Hu , and G. Gao . 2017. “Dynamics of Bacterial Abundance and the Related Environmental Factors in Large Shallow Eutrophic Lake Taihu.” Journal of Freshwater Ecology 32: 133–145. 10.1080/02705060.2016.1248506.

[ece371445-bib-0022] Gonzalez, J. M. , M. C. Portillo , P. Belda‐Ferre , and A. Mira . 2012. “Amplification by PCR Artificially Reduces the Proportion of the Rare Biosphere in Microbial Communities.” PLoS One 7: e29973. 10.1371/journal.pone.0029973. 22253843 PMC3256211

[ece371445-bib-0023] Hahn, M. W. , H. Lünsdorf , Q. Wu , et al. 2003. “Isolation of Novel Ultramicrobacteria Classified as Actinobacteria From Five Freshwater Habitats in Europe and Asia.” Applied and Environmental Microbiology 69: 1442–1451. 10.1128/AEM.69.3.1442-1451.2003. 12620827 PMC150105

[ece371445-bib-0024] Hu, Y. , G. Xie , X. Jiang , K. Shao , X. Tang , and G. Gao . 2020. “The Relationships Between the Free‐Living and Particle‐Attached Bacterial Communities in Response to Elevated Eutrophication.” Frontiers in Microbiology 11: 423. 10.3389/fmicb.2020.00423. 32269552 PMC7109266

[ece371445-bib-0025] Hugoni, M. , A. Vellet , and D. Debroas . 2017. “Unique and Highly Variable Bacterial Communities Inhabiting the Surface Microlayer of an Oligotrophic Lake.” Aquatic Microbial Ecology 79: 115–125. 10.3354/ame01825.

[ece371445-bib-0026] Ji, B. , J. Liang , Y. Ma , L. Zhu , and Y. Liu . 2019. “Bacterial Community and Eutrophic Index Analysis of the East Lake.” Environmental Pollution 252: 682–688. 10.1016/j.envpol.2019.05.138. 31185357

[ece371445-bib-0027] Kang, S. , and A. L. Mills . 2006. “The Effect of Sample Size in Studies of Soil Microbial Community Structure.” Journal of Microbiological Methods 66: 242–250. 10.1016/j.mimet.2005.11.013. 16423418

[ece371445-bib-0028] Lefranc, M. , A. Thénot , C. Lepère , and D. Debroas . 2005. “Genetic Diversity of Small Eukaryotes in Lakes Differing by Their Trophic Status.” Applied and Environmental Microbiology 71: 5935–5942. 10.1128/AEM.71.10.5935-5942.2005. 16204507 PMC1266003

[ece371445-bib-0029] Li, T. , S. Zhang , J. Hu , et al. 2023. “Soil Sample Sizes for DNA Extraction Substantially Affect the Examination of Microbial Diversity and Co‐Occurrence Patterns but Not Abundance.” Soil Biology and Biochemistry 177: 108902. 10.1016/j.soilbio.2022.108902.

[ece371445-bib-0030] Liu, J. , B. Fu , H. Yang , M. Zhao , B. He , and X.‐H. Zhang . 2015. “Phylogenetic Shifts of Bacterioplankton Community Composition Along the Pearl Estuary: The Potential Impact of Hypoxia and Nutrients.” Frontiers in Microbiology 6: 64. 10.3389/fmicb.2015.00064. 25713564 PMC4322608

[ece371445-bib-0031] Louca, S. , L. W. Parfrey , and M. Doebeli . 2016. “Decoupling Function and Taxonomy in the Global Ocean Microbiome.” Science 353: 1272–1277. 10.1126/science.aaf4507. 27634532

[ece371445-bib-0032] Magnuson, J. J. , B. J. Benson , and T. K. Kratz . 1990. “Temporal Coherence in the Limnology of a Suite of Lakes in Wisconsin, U.S.A.” Freshwater Biology 23: 145–159. 10.1111/j.1365-2427.1990.tb00259.x.

[ece371445-bib-0033] Mohapatra, M. , P. Behera , J. Y. Kim , and G. Rastogi . 2020. “Seasonal and Spatial Dynamics of Bacterioplankton Communities in a Brackish Water Coastal Lagoon.” Science of the Total Environment 705: 134729. 10.1016/j.scitotenv.2019.134729. 31838414

[ece371445-bib-0034] Newton, R. J. , S. E. Jones , A. Eiler , K. D. McMahon , and S. Bertilsson . 2011. “A Guide to the Natural History of Freshwater Lake Bacteria.” Microbiology and Molecular Biology Reviews 75: 14–49. 10.1128/mmbr.00028-10. 21372319 PMC3063352

[ece371445-bib-0035] Oksanen, J. , R. Kindt , P. Legendre , et al. 2009. “The Vegan Package.”

[ece371445-bib-0036] Orellana, R. , C. Macaya , G. Bravo , et al. 2018. “Living at the Frontiers of Life: Extremophiles in Chile and Their Potential for Bioremediation.” Frontiers in Microbiology 9: 2309. 10.3389/fmicb.2018.02309. 30425685 PMC6218600

[ece371445-bib-0037] Padilla, C. C. , S. Ganesh , S. Gantt , et al. 2015. “Standard Filtration Practices May Significantly Distort Planktonic Microbial Diversity Estimates.” Frontiers in Microbiology 6: 547. 10.3389/fmicb.2015.00547. 26082766 PMC4451414

[ece371445-bib-0038] Pagnier, I. , D. Raoult , and B. La Scola . 2011. “Isolation and Characterization of *Reyranella massiliensis* gen. nov., sp. nov. From Freshwater Samples by Using an Amoeba Co‐Culture Procedure.” International Journal of Systematic and Evolutionary Microbiology 61: 2151–2154. 10.1099/ijs.0.025775-0. 20889765

[ece371445-bib-0039] Penton, C. R. , V. V. S. R. Gupta , J. Yu , and J. M. Tiedje . 2016. “Size Matters: Assessing Optimum Soil Sample Size for Fungal and Bacterial Community Structure Analyses Using High Throughput Sequencing of Rrna Gene Amplicons.” Frontiers in Microbiology 7: 824. 10.3389/fmicb.2016.00824. 27313569 PMC4889595

[ece371445-bib-0040] Pernthaler, J. , and R. Amann . 2005. “Fate of Heterotrophic Microbes in Pelagic Habitats: Focus on Populations.” Microbiology and Molecular Biology Reviews 69: 440–461. 10.1128/mmbr.69.3.440-461.2005. 16148306 PMC1197807

[ece371445-bib-0041] Qin, B. , G. Gao , G. Zhu , et al. 2013. “Lake Eutrophication and Its Ecosystem Response.” Chinese Science Bulletin 58: 961–970.

[ece371445-bib-0042] Qin, B. , L. Yang , F. Chen , G. Zhu , L. Zhang , and Y. Chen . 2006. “Mechanism and Control of Lake Eutrophication.” Chinese Science Bulletin 51: 2401–2412.

[ece371445-bib-0043] Ranjard, L. , and A. Richaume . 2001. “Quantitative and Qualitative Microscale Distribution of Bacteria in Soil.” Research in Microbiology 152: 707–716. 10.1016/S0923-2508(01)01251-7. 11686384

[ece371445-bib-0044] Ren, Z. , X. Qu , W. Peng , Y. Yu , and M. Zhang . 2019. “Functional Properties of Bacterial Communities in Water and Sediment of the Eutrophic River‐Lake System of Poyang Lake, China.” PeerJ 7: e7318. 10.7717/peerj.7318. 31338262 PMC6628883

[ece371445-bib-0045] Richards, T. A. , A. A. Vepritskiy , D. E. Gouliamova , and S. A. Nierzwicki‐Bauer . 2005. “The Molecular Diversity of Freshwater Picoeukaryotes From an Oligotrophic Lake Reveals Diverse, Distinctive and Globally Dispersed Lineages.” Environmental Microbiology 7: 1413–1425. 10.1111/j.1462-2920.2005.00828.x. 16104864

[ece371445-bib-0046] Sepulveda, A. J. , J. Schabacker , S. Smith , R. Al‐Chokhachy , G. Luikart , and S. J. Amish . 2019. “Improved Detection of Rare, Endangered and Invasive Trout in Using a New Large‐Volume Sampling Method for eDNA Capture.” Environmental DNA 1: 227–237. 10.1002/edn3.23.

[ece371445-bib-0047] Shapiro, J. 1980. “The Importance of Trophic‐Level Interactions to the Abundance and Species Composition of Algae in Lakes.” In Hypertrophic ecosystems, edited by J. Barica and L. R. Mur , 105–116. Springer Netherlands. 10.1007/978-94-009-9203-0_12.

[ece371445-bib-0048] Shen, D. , K. Jürgens , and S. Beier . 2018. “Experimental Insights Into the Importance of Ecologically Dissimilar Bacteria to Community Assembly Along a Salinity Gradient.” Environmental Microbiology 20: 1170–1184. 10.1111/1462-2920.14059. 29393568

[ece371445-bib-0049] Tang, X. , G. Xie , J. Deng , et al. 2022. “Effects of Climate Change and Anthropogenic Activities on Lake Environmental Dynamics: A Case Study in Lake Bosten Catchment, NW China.” Journal of Environmental Management 319: 115764. 10.1016/j.jenvman.2022.115764. 35982565

[ece371445-bib-0050] Tang, X. , G. Xie , K. Shao , et al. 2020. “Contrast Diversity Patterns and Processes of Microbial Community Assembly in a River‐Lake Continuum Across a Catchment Scale in Northwestern China.” Environmental Microbiomes 15: 10. 10.1186/s40793-020-00356-9. PMC8066441 33902721

[ece371445-bib-0051] Turner, C. R. , M. A. Barnes , C. C. Y. Xu , S. E. Jones , C. L. Jerde , and D. M. Lodge . 2014. “Particle Size Distribution and Optimal Capture of Aqueous Macrobial eDNA.” Methods in Ecology and Evolution 5, no. 7: 676–684. 10.1111/2041-210X.12206.

[ece371445-bib-0052] Wilkinson, L. 2011. “ggplot2: Elegant Graphics for Data Analysis by Wickham, H.” Biometrics 67: 678–679. 10.1111/j.1541-0420.2011.01616.x.

[ece371445-bib-0053] Williamson, C. E. , W. Dodds , T. K. Kratz , and M. A. Palmer . 2008. “Lakes and Streams as Sentinels of Environmental Change in Terrestrial and Atmospheric Processes.” Frontiers in Ecology and the Environment 6: 247–254. 10.1890/070140.

[ece371445-bib-0054] Xie, G. , X. Tang , Y. Gong , K. Shao , and G. Gao . 2020. “How Do Planktonic Particle Collection Methods Affect Bacterial Diversity Estimates and Community Composition in Oligo‐, Meso‐ and Eutrophic Lakes?” Frontiers in Microbiology 11: 593589. 10.3389/fmicb.2020.593589. 33343534 PMC7746777

[ece371445-bib-0055] Xie, G. , X. Tang , K. Shao , G. Zhu , and G. Gao . 2021. “Bacterial Diversity, Community Composition and Metabolic Function in Lake Tianmuhu and Its Dammed River: Effects of Domestic Wastewater and Damming.” Ecotoxicology and Environmental Safety 213: 112069. 10.1016/j.ecoenv.2021.112069. 33631636

[ece371445-bib-0056] Xing, P. , Y. Tao , J. Luo , et al. 2020. “Stratification of Microbiomes During the Holomictic Period of Lake Fuxian, an Alpine Monomictic Lake.” Limnology and Oceanography 65: S134–S148. 10.1002/lno.11346.

[ece371445-bib-0057] Yan, Q. , J. C. Stegen , Y. Yu , et al. 2017. “Nearly a Decade‐Long Repeatable Seasonal Diversity Patterns of Bacterioplankton Communities in the Eutrophic Lake Donghu (Wuhan, China).” Molecular Ecology 26: 3839–3850. 10.1111/mec.14151. 28437572

[ece371445-bib-0058] Zhu, W. , L. Wan , and L. Zhao . 2010. “Effect of Nutrient Level on Phytoplankton Community Structure in Different Water Bodies.” Journal of Environmental Sciences 22: 32–39. 10.1016/s1001-0742(09)60071-1 20397384

[ece371445-bib-0059] Zwirglmaier, K. , K. Keiz , M. Engel , J. Geist , and U. Raeder . 2015. “Seasonal and Spatial Patterns of Microbial Diversity Along a Trophic Gradient in the Interconnected Lakes of the Osterseen Lake District, Bavaria.” Frontiers in Microbiology 6: 1168. 10.3389/fmicb.2015.01168. 26579082 PMC4623418

