# Supplementary material for: Effects of Filtration Volumes on Bacterial Diversity and Community Structure in Freshwater Lakes
Source: Ecol Evol. 2025 May 12;15(5):e71445. doi: 10.1002/ece3.71445 (PMC12068947; doi:10.1002/ece3.71445)

**TABLE S1** | Mean values of the main environmental parameters and bacterial abundance in the studied two lakes.

| **Physicochemical parameters** | **Lake Bosten** | **Lake Taihu** |
| --- | --- | --- |
| WT (℃) | 27.9 | 28.7 |
| DO (mg/L) | 7.73 | 5.58 |
| TDS (mg/L) | 941 | 297 |
| Sal (‰) | 0.74 | 0.21 |
| pH | 8.33 | 7.97 |
| TN (mg/L） | 0.80 | 2.86 |
| TDN (mg/L） | 0.73 | 0.48 |
| TP (mg/L） | 0.008 | 0.198 |
| TDP (mg/L） | 0.006 | 0.031 |
| Chl-a (ug/L) | 2.02 | 100.26 |
| TSS (mg/L) | 7.94 | 66.30 |
| LOI (mg/L) | 2.51 | 30.85 |
| ISS (mg/L) | 5.43 | 35.5 |
| Bacteria abundance (×10^6^) | 1.05 | 17.32 |

**FIGURE S1** | The schematic diagram of the experimental design, illustrating the collection of bacterial samples from water using different filtration volumes in Lake Bosten and Lake Taihu. Six filtration volumes (indicated by green boxes) were applied for Lake Bosten, while seven filtration volumes (indicated by red boxes) were applied for Lake Taihu.


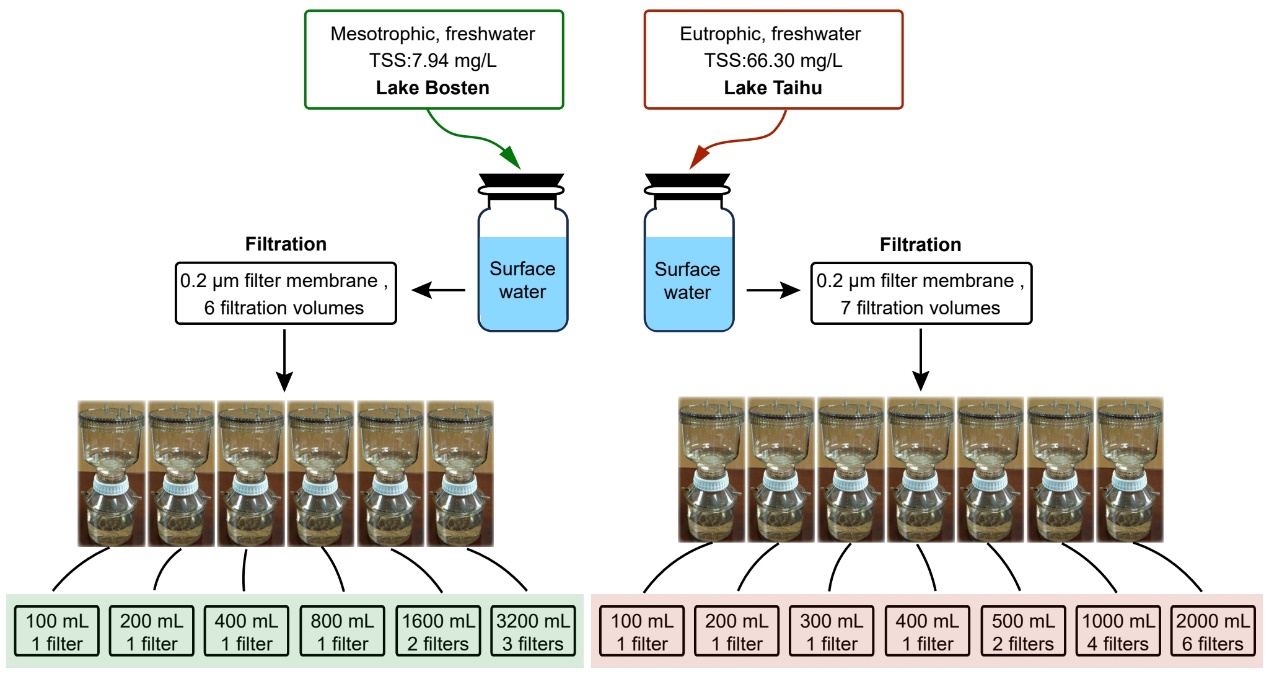


**FIGURE S2** | Map of China showing the location of the two lakes. Satellite images showing the sampling sites in Lake Bosten and Lake Taihu.


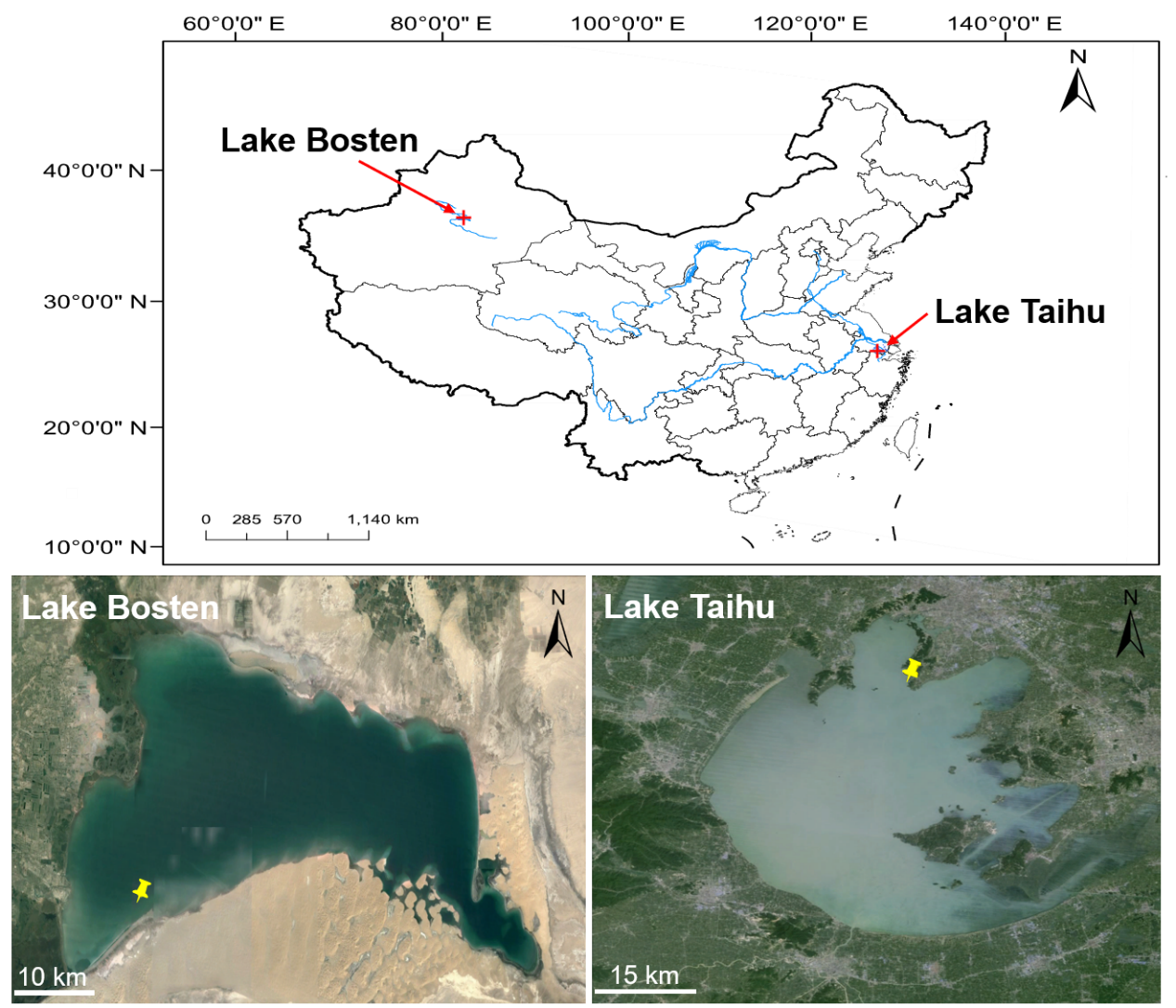


**FIGURE S3** | The rarefaction curves of the Shannon, Faith PD, and Richness indices for α diversity in Lake Bosten and Lake Taihu showed that, with increasing sequencing depth, the curves reached a saturation stage. This indicates that the populations captured most of the bacterial species present in each filtered volume sample.


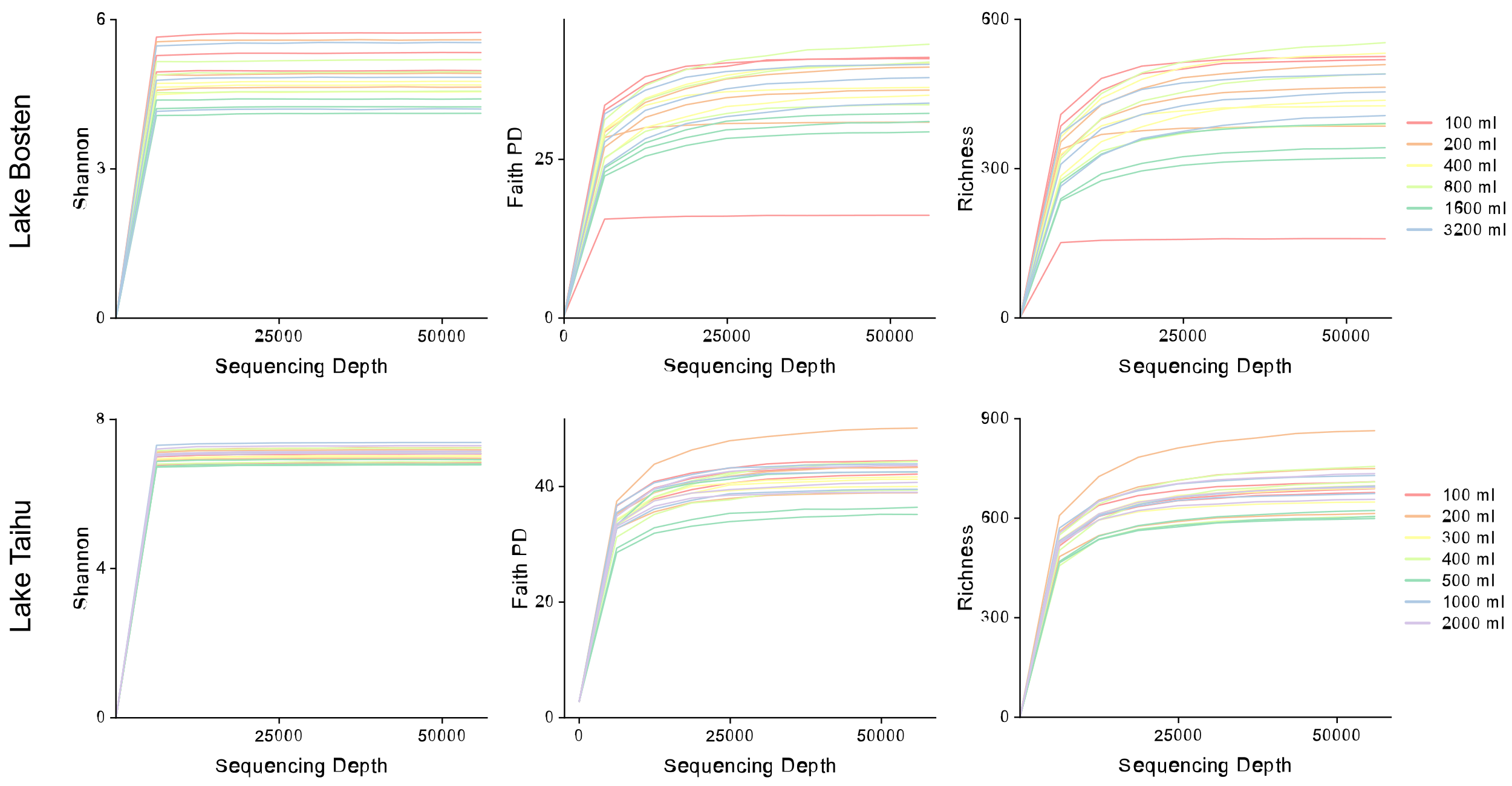

Supplement: Supplementary file 1 — Data S1 [file ECE3-15-e71445-s001.docx]
